# Syphilitic osteomyelitis in a patient with HIV and cognitive biases in clinical reasoning: A case report

**DOI:** 10.1097/MD.0000000000030733

**Published:** 2022-10-07

**Authors:** Kohei Kamegai, Shuhei Yokoyama, Shunichi Takakura, Yoshihiro Takayama, Soichi Shiiki, Hirofumi Koyama, Masashi Narita

**Affiliations:** a Division of Infectious Diseases, Department of Internal Medicine, Okinawa Prefectural Chubu Hospital, Uruma, Japan; b Disease Prevention and Control Center, National Center for Global Health and Medicine, Tokyo, Japan; c Division of Pathology, Okinawa Prefectural Chubu Hospital, Uruma, Japan; d Division of Infectious Diseases, Department of Internal Medicine, Okinawa Prefectural Nambu Medical Center & Children’s Medical Center, Haebaru, Japan.

**Keywords:** clinical reasoning, cognitive biases, gumma, HIV, syphilitic osteomyelitis

## Abstract

Diagnosing multifactorial, multidimensional symptoms unexplained by presumptive diagnosis is often challenging for infectious disease specialists.

**Patient concerns:**

We report a rare case of a 30-year-old Japanese bisexual man with a history of virally suppressed human immunodeficiency virus and syphilis infections who developed chest pain and an erosive lesion under the lower midline jaw.

**Diagnosis:**

Imaging examinations revealed erosive lesions on the sternum and left the ninth rib. Biopsy and polymerase chain reaction testing of sternal tissue specimens were noncontributory. However, due to elevated rapid plasma regain levels, a diagnosis of syphilitic osteomyelitis and gumma of the jaw was made.

**Interventions:**

The patient was treated with 5 weeks of intravenous ceftriaxone and then with 8 weeks of oral amoxicillin.

**Outcome:**

After the antibiotic treatment, bone pain disappeared. We conducted a literature review on syphilitic osteomyelitis, and all of the articles included were case reports. Approximately half of the 46 patients with syphilitic osteomyelitis had HIV coinfection, and 10 (22%) patients lacked signs of early syphilis. Given its rarity, clinical data to establish appropriate guidelines for diagnosing and treating syphilitic osteomyelitis are still lacking. Cognitive biases, such as anchoring, cognitive overload bias, and premature closure, may contribute to diagnostic delays.

**Lessons:**

In cases of idiopathic multiple bone lesions, syphilis must always be ruled out, and clinicians should guard against cognitive pitfalls when diagnosing rare diseases.

## 1. Introduction

Syphilis has various clinical manifestations and often occurs as a coinfection with other sexually transmitted infections, such as human immunodeficiency virus (HIV) infection.^[1]^ Osteomyelitis is a rare complication of syphilis observed in neonates born to infected mothers or in late-phase infections.^[2]^ We report the atypical clinical course of multifocal syphilitic osteomyelitis and gumma. Further, we discuss biases among clinicians when treating hard-to-solve cases.

## 2. Case presentation

A 30-year-old bisexual man presented at our outpatient clinic with worsening chest pain. He had a significant medical history of 2 previous syphilitic infections: a reddish rash on the face, trunk, and palms that appeared 2 years previously and was initially treated with amoxicillin 1500 mg/day, which, due to a drug-induced rash, was later switched to a 3-week course of doxycycline 200 mg/day, which was completed; and a few months later, the patient developed a generalized rash, which was treated with oral doxycycline 200 mg/day for 3 weeks. In both episodes, the rapid plasma regain (RPR) tests performed after treatment completion showed that posttreatment titers (RPR unit [RU]) decreased to less than a fourth of the pretreatment titers in the first and the second episode (RU 18.5–0.7 and 144.6–0.7, respectively). After 2 years, the patient had a HIV-1 and hepatitis B virus coinfection. His HIV infection was well controlled with dolutegravir, tenofovir alafenamide fumarate (TAF), and emtricitabine, and HIV-RNA was undetectable (<20 copies/mL) in the past 2 years. The latest hepatitis B surface antigen and hepatitis B virus DNA tests were negative, but the patient tested positive for both the hepatitis B surface antigen and hepatitis B core antibodies.

**Figure 1.**
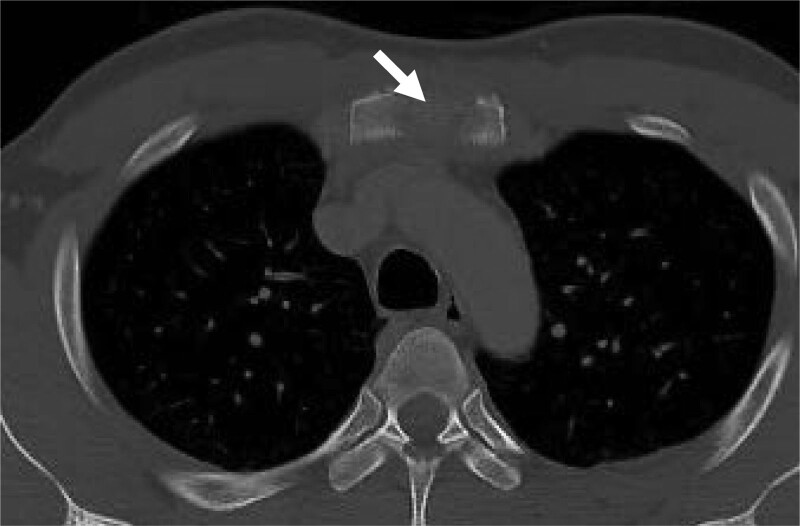
Osteolytic lesion in the sternum on plain computed tomography scan (white arrow).

Upon hospital arrival, the patient was alert and conscious, with stable vital signs (blood pressure, 102/60 mm Hg; respiratory rate, 20 breaths/min; heart rate, 100 beats/min; and body temperature, 37.9°C). He reported unprotected vaginal sexual intercourse with a female sex worker 1 month before the onset of chest pain but denied being homosexual or heterosexually active in recent years. Sternal and right sternoclavicular joint tenderness on physical examination was diagnosed as sternoclavicular arthritis for which the patient was prescribed painkillers. However, 2 months later, the patient presented with a limited range of motion and pain in the right upper arm. Plain chest computed tomography (Fig. 1) and magnetic resonance imaging (MRI) revealed erosive lesions on the sternum and the left ninth rib. A diagnostic sternal biopsy was performed, and the pathologist identified granulomas with caseous necrosis and a bacillus-like finding on Ziehl–Neelsen staining.

Microscopy and immunostaining were performed using the anti-*Treponema pallidum* rabbit polyclonal antibody; however, *T. pallidum* was not detected. Polymerase chain reaction tests of the sternal tissue and eschar for *Mycobacterium tuberculosis, Mycobacterium avium complex*, and *Mycobacterium intracellulare* were all negative, and the patient refused to undergo lumbar puncture. As sternal tuberculosis (TB) could not be ruled out, antitubercular chemotherapy was initiated. Given the possibility of interactions between TAF and rifampicin, TAF was replaced with tenofovir disoproxil fumarate.

**Figure 2.**
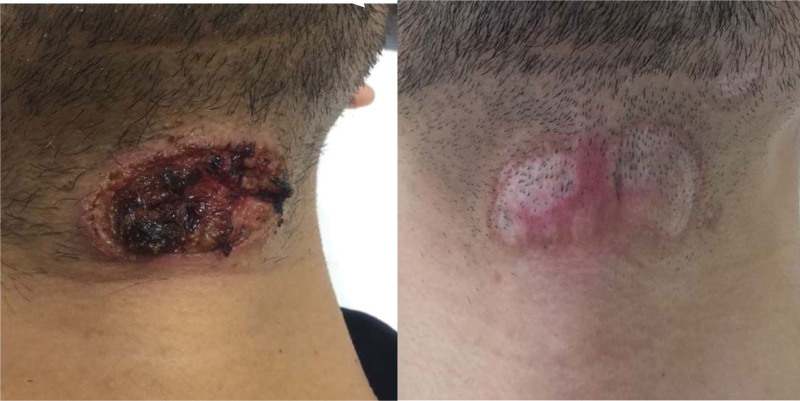
Clinical course of submandibular gumma before antibiotic treatment (left) and after 3 months of treatment (right).

**Figure 3.**
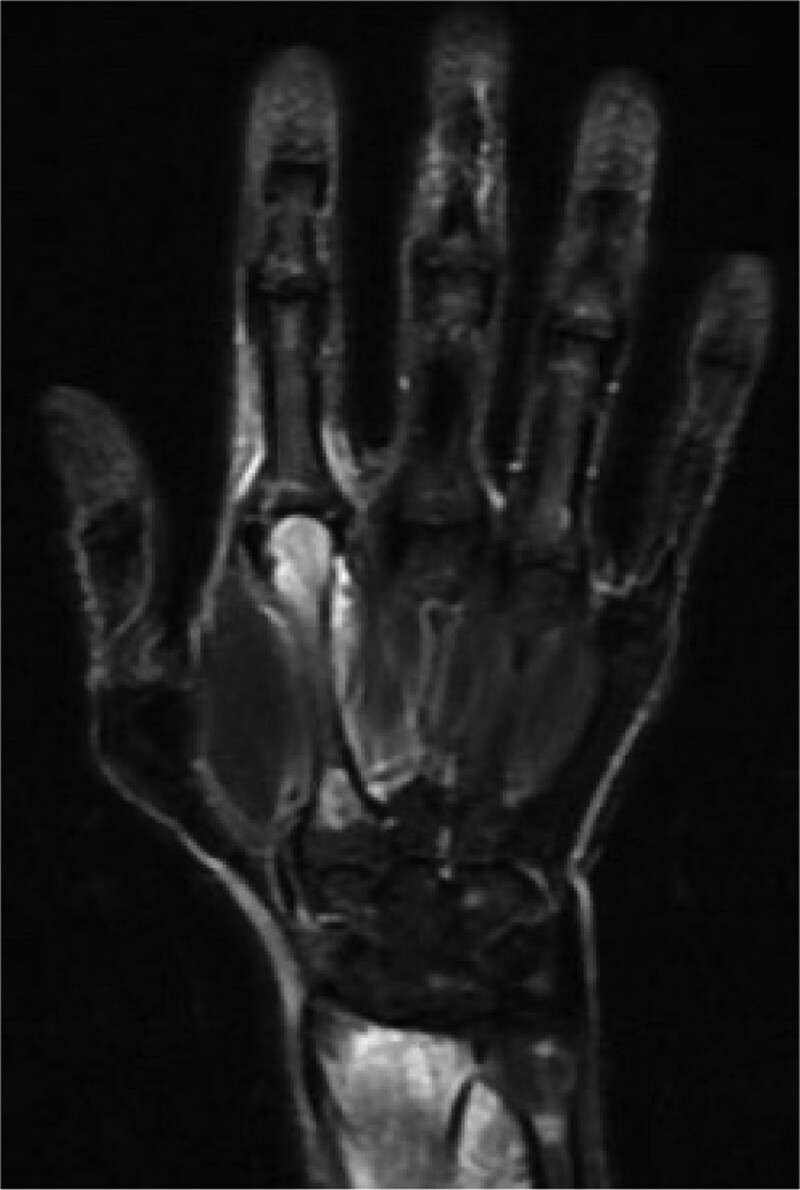
Plain magnetic resonance imaging (short T1-inversion recovery mode) of the right hand conducted 1 month before starting antibiotic treatment. The radius, ulna, and second metacarpal bone show high intensity.

One month later, the patient presented with a painless, erosive cutaneous lesion under the lower midline jaw (Fig. 2), which was diagnosed as pyoderma gangrenosum by a dermatologist. The patient was treated with oral prednisolone 5 mg/day. The patient complained of periarticular pain in the right wrist. On plain MRI (short T1-inversion recovery mode) of the right hand conducted 1 month before starting antibiotic treatment, the radius, ulna, and second metacarpal bone showed high intensity (Fig. 3). A week later, the submandibular lesion enlarged, and multiple erosive lesions appeared simultaneously all over the face and chest. The prednisolone dose was increased to 15 mg/day. Routine RPR screening performed 2 weeks later revealed a significant increase to 390 RU (baseline < 0.99). The sternal and mandibular lesions were negative for syphilis on immunohistochemistry and Warthin–Starry staining. The patient refused to undergo a bone biopsy. Repeated MRI scans showed additional abnormal signals in the ulna, radius, metacarpal bones, and proximal phalanx of the thumb. Owing to the elevated RPR, we tentatively diagnosed the patient with syphilitic gumma and osteomyelitis of the metacarpal bone, radius, and right upper ulna.

Given the patient’s amoxicillin allergy, intravenous ceftriaxone 2 g/day was administered and continued for 5 weeks after admission; subsequently, the gumma and bone pain nearly disappeared. After desensitization, we prescribed oral amoxicillin (250 mg/day, gradually increased to 1500 mg/day, without a generalized rash) for bone lesions that persisted on MRI. After discharge, amoxicillin 500 mg/three times daily for 8 weeks was continued until the pain completely disappeared. After the completion of antibiotic treatment, MRI showed abnormal signals in all the known lesions, but the sternal and mandibular lesions were negative for TB after culture for 8 weeks.

## 3. Discussion and Conclusions

The atypical clinical course of HIV infection makes it challenging to confirm a diagnosis based on differential diagnoses. To clarify the clinical features of this rare infection and identify factors that hinder accurate diagnosis, we conducted a literature search in PubMed using the following keywords: “syphilitic periostitis,” “syphilitic osteitis,” “syphilitic osteomyelitis,” and “bone syphilis.” We excluded non-English literature, articles published before 1983 (when HIV was discovered), congenital syphilis, and otologic syphilis. We identified 46 case reports (Tables 1 and 2), with nearly half of the cases involving HIV-positive patients and excluded case reports with missing data. The average time from onset to diagnosis was 7.0 (range, 1–36 weeks), which was unaffected by HIV positivity, a finding similar to that in our case. Thus, for most clinicians, syphilitic osteomyelitis is difficult to diagnose because of pathogen, host, and clinician factors.

**Table 1:** The characteristics of patients with syphilitic bone involvement.

Parameter	Total (N = 46)	(%)	HIV positive (N = 24)	(%)	Non-HIV (N = 22)	(%)
Male sex	40/46	(87)	24/24	(100)	16/22	(72.7)
Age, median (range)	41 (20–71)		40 (20–66)		47 (20-71)	
Clinical findings						
Bone pain	46/46	(100)	24/24	(100)	22/22	(100)
Early phase signs						
Recent history of genital ulcer	3/46	(6.5)	1/17	(5.9)	2/12	(16.7)
Rash	26/46	(56.5)	12/21	(57.1)	14/19	(73.7)
Lymphadenopathy	11/46	(23.9)	5/19	(26.3)	6/17	(35.3)
None of the above	10/46	(21.7)	7/24	(29.2)	3/22	(13.6)
Nontreponemal test (VDRL or RPR)	46/46	(100)	46/46	(100)	46/46	(100)
Sites of the affected bones						
Skull	25/46	(54.3)	14/24	(58.3)	11/22	(50.0)
Long bone of the limbs or fingers	22/46	(47.8)	15/24	(62.5)	7/22	(31.8)
Vertebra	7/46	(15.2)	3/24	(12.5)	4/22	(18.2)
Rib	4/46	(8.7)	3/24	(12.5)	1/22	(4.5)
Clavicle	2/46	(4.3)	1/24	(4.2)	1/22	(4.5)
Sternum	2/46	(4.3)	1/24	(4.2)	1/22	(4.5)
Pelvis	1/46	(2.1)	1/24	(4.2)	0/22	(0)
Histologic findings of bone biopsy						
*Treponema pallidum* detection	11/24	(45.8)	2/10	(20)	9/14	(64.3)
Average weeks from the onset to diagnosis (range)	7.0 (1–36)		7.3 (2–36)		6.7 (1–16)	

HIV = human immunodeficiency virus, RPR = rapid plasma regain, VDRL = Venereal Disease Research Laboratory.

**Table 2:** Treatment for syphilis with bone involvement.

Antibiotics regimen (including combination therapy)	No. of patients	(%)
Intramuscular benzathine penicillin	26/43	(60)
Once a week, for 9 wk	1/26	
Once a week, for 4 wk	1/26	
Once a week, for 3 wk	14/26	
Once a week, for 2 wk	5/26	
Once a week, for 1 wk	4/26	
Once a week, within 3 wk	23/26	
Unknown duration	1/26	
Combination with aqueous penicillin	8/26	
Intravenous aqueous penicillin	19/43	(44)
6 wk	2/19	
3 wk	3/19	
2 wk	10/19	
Unknown duration	4/19	
Oral doxycycline	3/43	(7)
16 wk	1/43	
6 wk	1/43	
4 wk	1/43	
Intravenous ceftriaxone	2/43	(5)
5 wk	1/2	
3 wk	1/2	
Oral azithromycin	2/43	(5)
10 wk	1/2	
2 wk	1/2	
Intravenous nafcillin	1/43	(2)
Unknown duration	1/1	

### 3.1. Pathogen

Early-stage syphilis typically presents with a rash, local lymphadenopathy, or genital lesions, which were absent in the present case. In our literature review, only 10 of the 46 patients (22%) showed no signs of early syphilis prior to the onset of bone lesions, which supports our inference that osteomyelitis without early syphilitic symptoms is an atypical presentation of syphilis.

### 3.2. Host

Our literature review showed that compared to patients without HIV, patients with HIV infection were less likely to manifest early signs of syphilis (13.6% vs 29.2%), which is consistent with the presentation in our case. Reinfection with syphilis is more likely to be asymptomatic than the initial infection.^[3]^ As this was the third syphilitic infection with an HIV coinfection in our patient, the lack of early symptoms is plausible because impaired cell-mediated immune mechanisms in HIV-positive patients^[4]^ may mask the early signs.

### 3.3. Clinician bias

From a retrospective viewpoint, we identified a cognitive bias in our thought processes. Cognitive bias corresponds to data based on past experiences or current situations.^[5]^ Errors caused by cognitive biases play a role in more than 50% and 83% of diagnostic errors in ambulatory care and those reported by physicians, respectively.^[5]^ We discussed three significant cognitive biases: anchoring, cognitive overload, and premature closure.

Anchoring bias tends not to reconsider the initial diagnosis despite conflicting information.^[6]^ Particularly, after initiating treatment based on the diagnosis, there is a robust tendency to adhere to the presumed diagnosis. In the present case, the pathological findings led us to initiate treatment for bone TB, although the tissue polymerase chain reaction result was negative. After the treatment, another pathologist retrospectively determined that the Ziehl–Neelsen staining result was a false positive.

Cognitive overload bias occurs when the clinician’s cognitive abilities are overwhelmed by excessive information.^[6]^ We believe that most clinicians are unfamiliar with syphilis osteomyelitis without signs of early syphilis. This atypical clinical course and the masquerading pathological findings may have overwhelmed the clinicians.

Premature closure occurs when a clinician confirms a diagnosis with inadequate information.^[6]^ In this case, the working diagnosis of bone TB did not explain all the findings at several points, such as the rapid progression of bone lesions. The patient was initially considered to have presented too early to have syphilitic osteomyelitis because he reported having unprotective heterosexual intercourse 1 month before symptom onset. However, clinicians have failed to consider the possibility of unprotected sexual activity between the second and third syphilis infections, thereby underestimating the risk of syphilis reinfection. As determined in an observational study, repeated syphilis episodes do not reduce the risk of subsequent syphilis infection.^[7]^

Guidelines for the diagnosis of syphilitic osteomyelitis have not been established. In this case, the bone biopsy did not detect any evidence of syphilis. Multifocal osteomyelitis with gumma was strongly suspected because, (1) the non-treponemal test result was positive and (2) the bone pain and ulcerative skin lesion disappeared with antimicrobial agents that are effective in syphilis. Without the supporting results of bone biopsy, a positive non-treponemal test alone does not prove that the bone lesions were caused by syphilis. All 46 patients identified in this literature review complained of bone pain in the affected area, and bone biopsy was performed in approximately half of them (24/46 cases, 52%). *T. pallidum* was identified in 46% (11/24) of the patients. Moreover, in HIV-infected patients, the detection rate of *T. pallidum* was only 20% (2/10), which was lower than that in non-HIV-infected patients (64.3%, 9/14). We believe that a bone biopsy should be performed when bone syphilis is suspected; however, the sensitivity of a bone biopsy may be lower in patients with HIV.

The Centers for Disease Control and Prevention (CDC) recommends 3 doses of 2.4 million units of intramuscular benzathine penicillin each at 1-week intervals for late latent syphilis,^[8]^ but the guideline does not mention a treatment for bone lesions. In the literature review, 60% (26/43) of the patients were treated with regimens that included intramuscular injection of benzathine penicillin. In Japan, intramuscular benzathine penicillin was approved by the Ministry of Health, Labour, and Welfare in 2021 but was unavailable in this case. The other drugs included intravenous injections of aqueous penicillin (44%; 19/43), doxycycline (7%; 3/43), ceftriaxone (5%; 2/43), azithromycin (5%; 2/43), and nafcillin (2%; 1/43). Furthermore, the appropriate duration of antimicrobial therapy for the treatment of syphilitic osteomyelitis is unknown. This literature review showed that 23/26 (88%) patients completed the CDC’s recommended regimen for late-stage syphilis. In our case, considering the possible risk of an allergic reaction to penicillin, ceftriaxone was administered for 5 weeks, followed by intravenous amoxicillin (500 mg three times a day) for 8 weeks until the pain disappeared completely. The long treatment duration may have been due to the unavailability of intramuscular benzylpenicillin, which may have affected treatment efficacy.

This study had some limitations. The review is limited to English-language literature wherein HIV status is specified, and because syphilitic osteomyelitis is a rare disease, the data are insufficient. Moreover, most cases were from Asia, Europe, and the United States, while reports from most developing countries were not included. To the best of our knowledge, there are no reports from the African continent, the region with the most significant number of people with HIV. Sometimes, syphilis could prove to be challenging to diagnose in developing countries, and many cases are not correctly diagnosed owing to a lack of infrastructure.

Given the limitations, it is almost impossible to conduct a cohort study on syphilitic osteomyelitis. However, our report describes the characteristics of syphilitic osteomyelitis in HIV patients, factors that delay diagnosis, and the prejudices and pitfalls that clinicians tend to experience. As syphilis is a systemic infection and multisystemic disease, its diagnosis is sometimes difficult even for well-trained, experienced clinicians. Syphilis should always be considered in the differential diagnosis of patients with symptoms of unknown origin such as bone pain or skin lesions.

The diagnosis of syphilitic osteomyelitis in patients with HIV may prove more challenging, especially for less experienced clinicians. Although conclusive diagnosis of multiple bone lesions may not be possible, syphilis must always be ruled out. Furthermore, clinicians should exercise efforts to avoid cognitive pitfalls when diagnosing rare diseases.

## Acknowledgments

We are grateful to the patients for their collaboration in this study. We also thank Katsuya Chinen, MD, PhD, for providing valuable comments on the pathological findings.

## Author contributions

**Conceptualization:** Kohei Kamegai.

**Data curation:** Kohei Kamegai.

**Formal analysis:** Kohei Kamegai.

**Investigation:** Kohei Kamegai, Masashi Narita.

**Methodology:** Kohei Kamegai.

**Project administration:** Kohei Kamegai, Masashi Narita.

**Resources:** Kohei Kamegai.

**Supervision:** Hirofumi Koyama, Masashi Narita, Shuhei Yokoyama, Shunichi Takakura, Soichi Shiiki, Yoshihiro Takayama.

**Validation:** Kohei Kamegai.

**Visualization:** Kohei Kamegai.

**Writing – original draft:** Hirofumi Koyama, Kohei Kamegai.

**Writing – review & editing:** Kohei Kamegai, Masashi Narita, Shuhei Yokoyama, Shunichi Takakura, Soichi Shiiki, Yoshihiro Takayama.
